# Non-invasive biomarkers for diagnosis and monitoring of primary mitochondrial diseases

**DOI:** 10.1007/s00415-026-13794-1

**Published:** 2026-04-10

**Authors:** Ignazio Giuseppe Arena, Shamini Saravanabavan, Rita Horvath, Jelle van den Ameele

**Affiliations:** 1 Department of Clinical Neurosciences, University of Cambridge, Cambridge, UK; 2 MRC Mitochondrial Biology Unit, University of Cambridge, Cambridge, UK

**Keywords:** Mitochondrial disease, Biomarkers, Functional endpoints, Wearable devices, Digital health technologies, Phenotyping, Neuroimaging, Magnetic resonance imaging, Positron emission tomography, Precision medicine, Clinical trials

## Abstract

Primary mitochondrial diseases (PMDs) represent a clinically and genetically heterogeneous group of disorders characterized by impaired oxidative phosphorylation and multisystem involvement, commonly affecting the nervous system. As therapeutic development accelerates, there is a growing need for robust biomarkers capable of supporting diagnosis, stratifying patient subgroups, monitoring disease progression, and providing sensitive pharmacodynamic readouts for clinical trials. This review summarizes recent advances in three major non-invasive biomarker domains relevant to PMDs: circulating serum and molecular biomarkers, functional and digital endpoints, and neuroimaging modalities. Circulating markers, such as FGF21, GDF15, NfL, and NAD⁺-related signatures, have each been proposed for diagnosis and to follow disease progression, while multi-omics approaches are paving the way toward integrated molecular phenotyping. Digital health technologies, including accelerometry and gait analytics, enable objective quantification of real-world functional impairment, although disease-specific validation remains an unmet need. Neuroimaging offers mechanistic insights through metabolic (MRS, CEST), perfusion (ASL), and molecular modalities (mitochondrial PET tracers). Cutting-edge tools, such as Multi-Spectral Optoacoustic Tomography (MSOT), Raman spectroscopy, and Near-Infrared Spectroscopy (NIRS), promise real-time or spatially resolved assessment of mitochondrial function. Together, these developments outline multidimensional biomarker approaches for PMDs, with the potential to directly measure target engagement and clinically meaningful phenotypes in future therapeutic trials. Future progress will depend on longitudinal validation, harmonized acquisition protocols, and the integration of multimodal platforms to support upcoming therapeutic trials and precision medicine strategies.

## Introduction

Primary mitochondrial diseases (PMDs) comprise a clinically heterogeneous group of disorders caused by pathogenic variants in mitochondrial or nuclear genes, resulting in impaired oxidative phosphorylation (OXPHOS). With an estimated prevalence of approximately 1 in 4,300 individuals [1], PMDs represent one of the most common inherited metabolic disorders. They may present with a wide range of neurological and multisystem manifestations, each posing significant challenges for diagnosis and management [2].

Despite major advances in genetic diagnosis [3] and the understanding of mitochondrial disease pathophysiology [4, 5], effective therapeutic options remain limited [6]. To date, only three drugs have received regulatory approval for the treatment of specific forms of PMDs: idebenone for Leber’s Hereditary Optic Neuropathy (LHON) [7] in Europe, and more recently, elamipretide for Barth Syndrome [8] and deoxy-pyrimidines for Thymidine kinase 2 (TK2)-related mtDNA depletion syndrome [9, 10] in the United States. However, most mitochondrial syndromes still lack disease-modifying interventions [6, 10–13], and an increasing number of emerging therapeutic strategies underscore the urgent need for reliable and clinically meaningful biomarkers [14, 15].

Biomarkers, defined as measurable indicators of normal biological processes, pathogenic mechanisms, or responses to therapeutic interventions [16], hold particular promise in PMDs. Given the pronounced clinical heterogeneity and diagnostic complexity of these disorders, robust biomarkers with direct relevance for the underlying pathophysiology are essential to improve diagnostic accuracy and patient stratification. There is an urgent need for biomarkers to monitor disease progression, predicting acute neurological events such as stroke-like episodes or sudden visual loss, and objectively assessing treatment responses or target engagement in experimental medicine studies and clinical trials [17].

Recent years have witnessed progress in biomarker discovery, driven by targeted and untargeted omics approaches, advances in imaging, and in digital and functional assessments tailored to specific phenotypes [18]. However, despite their promise, most still require rigorous validation.

The translation of candidate biomarkers into clinical practice or trials requires a progressive stepwise process of analytical and clinical validation followed by regulatory qualification within frameworks established by agencies, such as the European Medicines Agency (EMA), the U.S. Food and Drug Administration (FDA), or the UK Medicines and Healthcare products Regulatory Agency (MHRA). Biomarkers must demonstrate robust validity, including reproducibility, sensitivity and specificity and must be clearly defined within a specific “context of use” (e.g., diagnostic, monitoring, pharmacodynamic (response), predictive, etc.)[19]. This process typically requires evidence derived from multiple independent cohorts, standardized analytical methods, and harmonized protocols across centers. These challenges are amplified in rare diseases such as PMDs due to limited patient populations and heterogeneous disease progression [20].

Consequently, trial outcome measures have largely relied on established generic clinical or functional endpoints, rather than biomarkers that provide direct and short-term measures of target engagement or mitochondrial function. For example, studies on elamipretide in Barth Syndrome relied on echocardiography; idebenone in LHON stabilized and restored visual acuity; and deoxynucleoside supplementation in TK2-deficiency improved respiratory function, the 6-min walk test and reduced GDF15 levels [7–9].

**Fig. 1:**
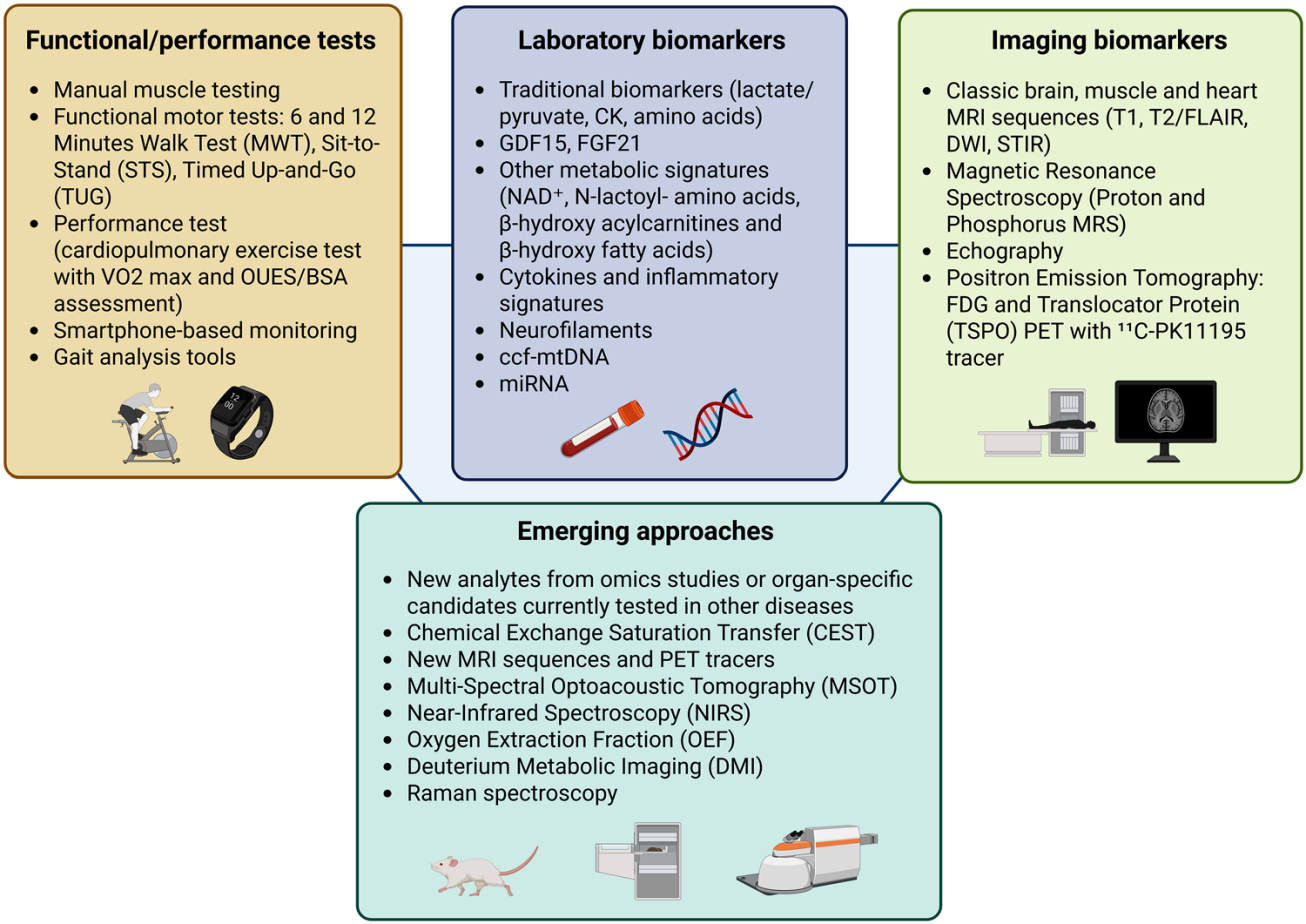
Non-invasive monitoring in primary mitochondrial disorders (PMDs). Biomarkers and tests for three major non-invasive monitoring domains in PMDs include functional and performance motor tests, laboratory biomarkers, and imaging biomarkers. Emerging approaches include promising examples of biomarkers that have been tested in model systems or patients with other neurodegenerative or neuromuscular conditions with potential future applications for PMDs though further studies are still needed. *DWI* Diffusion-Weighted Imaging, *FDG* Fluorodeoxyglucose, *FGF21* Fibroblast Growth Factor 21, *GDF15* Growth Differentiation Factor 15, *MRI* Magnetic Resonance Imaging, *MRS* Magnetic Resonance Spectroscopy, *OUES/BSA* Oxygen Uptake Efficiency Slope/Body surface Area, *PET* Positron Emission Tomography, *VO2* max Maximal Oxygen Consumption

This review provides an overview of the most relevant current and emerging clinical biomarkers for PMDs, highlighting their applicability and future utility for longitudinal disease monitoring. We summarize advances in laboratory biomarkers, neuroimaging modalities, and digital endpoints, and discuss possible future directions, such as molecular imaging and integrative multi-omics (Fig. 1, Table 1). As novel therapies advance toward clinical translation, the development and validation of sensitive, specific, and reproducible biomarkers will be critical to enable early diagnosis, optimize patient stratification, and reliably evaluate therapeutic efficacy.

**Table 1:** Tentative classification of non-invasive biomarkers, according to both their clinical role (diagnostic, monitoring, pharmacodynamic) and their current level of validation

Clinical application in PMDs	Exploratory	Partially validated	Clinically actionable
Diagnostic	Cardiopulmonary exercise testing with Oxygen Uptake Efficiency Slope/Body Surface Area (OUES/BSA)		
	miRNA; ‘omics signatures	Gelsolin; NAD^+^; CHIT1	Lactate; Pyruvate; Lactate/Pyruvate ratio; CK; amino acids; citrulline; Thd; dUrd; GDF15; FGF21
	Raman spectroscopy; Near Infrared Spectroscopy (NIRS) with vascular occlusion test	^31^P-MRS; PET-FDG	CT and MRI standard sequences (T2/FLAIR, DWI); ^1^H-MRS (with lactate peak)
Monitoring	Smartphone and/or wearable-based monitoring tools; SV95C; Cardiopulmonary exercise testing with Oxygen Uptake Efficiency Slope/Body Surface Area (OUES/BSA)	Gait analysis metrics (e.g., GAITrite)	Traditional functional/performance tests (6- and 12-min walk test, 30-s Sit-to-Stand test, Timed Up and Go test, Cardiopulmonary exercise testing with VO2 max)
	miRNA; cytokines; ‘omics signatures	FGF21; NfL; pNfH; ccf-mtDNA	Nucleosides (dThd, dUrd); GDF15
	MSOT; Near Infrared Spectroscopy (NIRS); Chemical Exchange Saturation Transfer (CEST); Raman spectroscopy	TSPO PET; ^31^P-MRS; ^1^H-MRS (with lactate peak)	
Pharmacodynamic (response biomarker)	SV95C; digital health-based composite endpoints		Nucleosides (dThd, dUrd); GDF15*
	‘omics signatures		
	PET with new mitochondrial tracers (^18^F-BCPP-EF, TPSO); Oxygen Extraction Fraction (OEF); Near Infrared Spectroscopy (NIRS) with vascular occlusion test; MSOT; Raman spectroscopy; MRI with Deuterium Metabolic Imaging	^31^P-MRS	

“Exploratory” biomarkers refer to early-stage candidates supported primarily by preclinical studies or small clinical cohorts. “Partially validated” biomarkers have shown reproducible associations with disease biology or severity in independent cohorts but lack widespread clinical implementation or regulatory qualification. “Clinically actionable” biomarkers are currently used in clinical diagnostic workflows or patient management

*ccf-mtDNA* Circulating Cell-Free mtDNA; *CK* Creatine kinase; *FGF21* Fibroblast Growth Factor 21; *GDF15* Growth Differentiation Factor 15; *DWI* Diffusion-Weighted Imaging; *FDG* Fluorodeoxyglucose; *FLAIR* Fluid-Attenuated Inversion Recovery; *MRI* Magnetic Resonance Imaging; *MRS* Magnetic Resonance Spectroscopy; *MSOT* Multi-Spectral Optoacoustic Tomography; *NfL* Neurofilament Light Chains; *OEF* Oxygen Extraction Fraction; *PET* Positron Emission Tomography; *SV95C* Stride Velocity 95th Centile; *TSPO* Translocator protein; *VO2 max* Maximal Oxygen Consumption

*In specific diseases (Thymidine Kinase 2 deficiency and Mitochondrial Neurogastrointestinal Encephalopathy)

## Functional assessments: from motor tests to digital monitoring

Fatigue is one of the most prevalent and disabling symptoms reported by individuals with PMDs, profoundly affecting daily functioning and quality of life and frequently co-occurring with muscle weakness, pain, and reduced mobility [21]. Consequently, the evaluation of fatigue and motor performance is central to both clinical monitoring and assessment of therapeutic efficacy.

Traditional functional assessments, including manual muscle testing, functional motor scales, cardiopulmonary exercise test and timed performance measures, have been adapted from other neuromuscular diseases and validated in PMDs [22–26]. However, these tools are often limited by inter-rater variability, ceiling and floor effects, motivational dependence, and insufficient sensitivity to capture subclinical or slowly progressive changes, particularly in heterogeneous and multisystem disorders [27].

Digital health technologies (DHTs) have emerged as a promising avenue to overcome these limitations. Wearable and non-wearable sensors enable objective and continuous quantification of motor and physiological function, either during structured in-clinic assessments or remotely during everyday activities [28]. Their successful implementation in other neuromuscular diseases, such as Duchenne muscular dystrophy, where digital gait biomarkers (e.g., Stride Velocity 95th Centile) have been qualified by regulatory agencies, provides a compelling precedent for their potential application in PMDs [29].

Initial investigations in PMDs indicate that wearable-based assessments are both feasible and informative. One of the earliest cross-sectional studies employing actigraphy demonstrated significantly reduced habitual physical activity in PMD patients, with daily step count inversely correlating with disease burden measured by the Newcastle Mitochondrial Disease Adult Scale (NMDAS) [30]. Complementary work using portable gait analysis systems revealed discrete gait abnormalities in adults with m.3243A > G and m.8344A > G variants, even among mildly affected individuals, underscoring the sensitivity of accelerometry-based tools to detect subtle motor impairments [31]. In pediatric cohorts, accelerometry similarly distinguished affected children from age- and sex-matched controls across domains of physical activity [32]. More advanced gait quantification platforms have also shown excellent reproducibility. The GAITRite electronic walkway has been validated in adults with the m.3243A > G variant, demonstrating high intra-class correlation coefficients across walking conditions and significant correlations between gait metrics and NMDAS scores [33]. Nonetheless, the responsiveness of wearable-derived outcomes remains an area of active investigation: in a longitudinal study assessing bezafibrate treatment, no significant change was observed in accelerometry-based activity levels, despite biochemical improvement, illustrating both the promise and challenges of incorporating digital endpoints into PMD trials [34].

Collectively, wearable and portable technologies can provide objective, reproducible, and clinically meaningful assessments of motor function and physical activity in PMDs. To advance their integration into clinical research, large-scale longitudinal studies, harmonized protocols, and disease-specific validation frameworks are needed. Future work should aim to develop multimodal digital biomarker profiles—combining movement data with cardiorespiratory, sleep, and fatigue-related metrics—to comprehensively capture the complex and fluctuating functional impairments that characterize PMDs.

## Laboratory biomarkers: untargeted and targeted approaches

Given the predominant bioenergetic impairment and frequent muscle involvement in PMDs, traditional CSF and plasma analytes, such as lactate, lactate/pyruvate ratio, amino acids, and creatine kinase (CK), have long served as adjunct biochemical indicators to support diagnosis, alongside muscle biopsy [35–37]. However, extensive clinical experience has demonstrated limited sensitivity and specificity for both diagnostic and longitudinal monitoring, as they may also reflect secondary mitochondrial dysfunction in a range of conditions including hypoxia, sepsis, seizures, or intense exercise [38, 39]. Therefore, their diagnostic utility has further declined with the widespread implementation of next-generation sequencing [3].

Omics-based approaches have gained considerable traction over the past decade, deepening mechanistic understanding of PMD pathophysiology and providing candidates for subsequent targeted evaluation [40]. Early transcriptomic and metabolomic work in mitochondrial disease models has shown profound oxidative stress remodeling converging on the induction of fibroblast growth factor 21 (FGF21), a metabolic cytokine secreted by stressed muscle fibers [41]. FGF21 has since been validated as a biomarker for mitochondrial myopathies, especially those associated with impaired mitochondrial DNA maintenance or translation [42–48]. Nonetheless, FGF21 can also be elevated in other conditions, including liver diseases and other metabolic neuromuscular disorders. Growth differentiation factor 15 (GDF15), another stress-responsive cytokine, has subsequently emerged as a complementary marker, initially as differentially expressed gene in muscle from patients with TK2-deficiency [49] and later also associated with other PMDs [50, 51]. Although its diagnostic specificity is limited by elevations in cancer, inflammation, and other metabolic and neuromuscular conditions [52], GDF15 has demonstrated promise particularly in TK2 deficiency, where it correlates with disease severity and response to nucleoside supplementation [53, 54]. GDF15 detection in saliva might enable minimally invasive monitoring [55], and integrating measurements with plasma gelsolin was shown to improve diagnostic accuracy [56].

Proteomic profiles from a cohort of mitochondrial patient fibroblasts pointed out the expression of 5 proteins (GPX4, ORF4L1, MOXD1, MSRA, and TMED9), as putative novel biomarkers [57]. Hypocitrullinemia was found in MT-ATP6–related conditions, while tyrosine was shown to be elevated in deoxyguanosine kinase deficiency [58–60]. Metabolomic and multi-omics profiling of urine samples in carriers of the common MELAS m.3243A > G variant [61] or in patients with progressive external ophthalmoplegia [62], have demonstrated systemic molecular alterations, supporting the sensitivity of urinary profiling in PMDs. Metabolomics have also been applied in plasma from Maternally Inherited Diabetes and Deafness (MIDD) patients [63], and more recently, in Mitochondrial Neurogastrointestinal Encephalomyopathy (MNGIE) [64], showing disease-specific patterns of organ involvement and supporting development of integrated multi-omics signatures rather than reliance on single biomarkers for future clinical trials.

Circulating signatures of NADH-driven reductive stress have been associated with disease burden in m.3243A > G, including well-established (lactate, alanine, GDF15, α-hydroxybutyrate) as well as new groups of analytes such *N*-lactoyl-amino acids, β-hydroxy acylcarnitines, and β-hydroxy fatty acids [65]. Reduced NAD⁺ levels have been proposed as a biomarker with direct relationship to the underlying biochemical OXPHOS defect in PMDs, and are being explored as a therapeutic target, for example in trials of niacin supplementation [42, 66]. Among the other metabolites with direct relevance for the underlying genetic or biochemical defect, thymidine/deoxyuridine levels are well-established as diagnostic biomarkers in MNGIE due to thymidine phosphorylase deficiency [67], where they also act as response biomarkers to measure therapeutic efficiency of liver or bone marrow transplantation [68, 69], and novel advanced therapies [70–72].

Whereas the aforementioned biomarkers broadly reflect primary mitochondrial dysfunction and may reflect tissue-specific disease phenotypes, several other biomarkers are more broadly associated with organ damage and acute disease activity in other conditions. Several of these have also been explored in PMDs. Neurofilament light chain (NfL), widely used in neurodegenerative disorders, has shown promise as a marker of neuroaxonal injury in PMDs in serum and in the cerebrospinal fluid, where it correlates with clinical severity in MELAS [73–77]. Elevated circulating cell-free mitochondrial DNA (ccf-mtDNA) may act as an indicator of mitochondrial stress during acute MELAS episodes [78, 79]. Chitotriosidase (CHIT1), historically used in lysosomal storage diseases, was found to be elevated in PMDs versus other neuromuscular diseases (NMDs) and healthy controls [80]. Finally, recent studies have identified inflammatory activation and upregulation of interferon-stimulated genes in pediatric PMD cohorts [81, 82], and have explored changes in cytokines [83] or circulating microRNAs as possible stable, disease-related indicators in MNGIE [84], MELAS [85], or patients with PMDs and sensorineural hearing loss [86]. At present, most of these remain adjunctive tools for diagnosis, stratification, and trial readiness, pending longitudinal validation. A summary of the main circulating biomarkers that have been tested in patients with mitochondrial disease, is provided in Table 2.

**Table 2:** List of the main circulating analytes/biomarkers tested in Primary Mitochondrial Diseases (PMDs)

Organ	Analyte/Biomarker	Mitochondrial condition(s)	Findings and applications/limits	References
Blood	Lactate* Pyruvate*; Lactate/pyruvate ratio	Tested in different PMDs	Elevated in different PMDs but overall limited diagnostic sensitivity/specificity. Pyruvate and lactate/pyruvate could be useful as a complementary biomarker in Pyruvate Dehydrogenase and Pyruvate Carboxylase deficiencies	Paredes-Fuentes et al. [39]
	Creatine kinase (CK)	Tested in different PMDs	Elevated in myopathic forms, but limited diagnostic sensitivity/specificity, apart from specific contexts (TK2 deficiency)	Debray et al. [36]
	Amino acids (alanine*, aspartate)	Tested in different PMDs	Occasionally elevated, especially in early onset forms. Overall low sensitivity and specificity	Paredes-Fuentes et al. [39]
	Citrulline	ATP6-related disorders	Reduced in ATP6-related disorders	Li et al., [58]; Carli et al. [59]
	Thymidine (Thd), deoxyuridine (dUrd)	MNGIE	Thd and dUrd levels are useful in MNGIE patients	Hirano et al. [67]
	FGF21	Tested in different PMDs, mostly mitochondrial myopathies	Elevated in mtDNA maintenance/translation defects. Validated as biomarker of mitochondrial myopathy but low diagnostic specificity	Forsstrom et al. [42]
	GDF15	Tested in different PMDs, particularly TK2 deficiency	Elevated in PMDs but low specificity; correlates with disease severity and decreases with therapy in TK2 deficiency	Tsygnkova et al. [52], Bermejo-Guerrero et al. [53]
	Gelsolin	Tested in a cohort of different PMDs, including CPEO, MELAS	Reduced. Evaluated in association with GDF15, can improve diagnostic accuracy	Penas et al. [56]
	NADH-related reductive stress markers and NAD⁺	Tested in a cohort of different PMDs, particularly with mitochondrial myopathy	Circulating signatures correlate with disease burden; Reduced NAD⁺, explored as biomarker and target in niacin trial	Pirinen et al., [66]
	Neurofilament light chain (NfL)*	Tested in different disorders, particularly in MELAS	Serum NfL elevated in neuroaxonal injury; biomarker potential. In CSF correlated with disease severity	Varhaug et al. [74]; Sofou et al. [73]
	Phosphorylated neurofilament heavy chain (pNfH)	LHON	Suggested as marker of axonal degeneration after visual loss function	Guy et al. [75]
	Interferon-stimulated genes	Tested in a cohort of pediatric PMDs	Upregulated; may reflect immune dysregulation	Keshavan et al. [81]
	Cytokines	PMDs	May reflect inflammatory activation, cytokine alterations. Need further validation	Primiano et al. [83]
	Chitotriosidase (CHIT1)	PMDs	Elevated; candidate biomarker for PMDs	Foerster et al. [80]
	Arachidonic acid metabolism, bile acid biosynthesis	MNGIE	Significant upregulation; disease-specific metabolic pattern, potentially specific for MNGIE	Bax et al. [64]
	miRNA	MNGIE	miR-192-5p, miR-193a-5p, miR-194-5p, miR-215-5p and miR-34a-5p in plasma; miR-192-5p, miR-194-5p, miR-34a-5p: potentially specific for MNGIE	Mencias et al. [84];
		PMD patients with sensorineural hearing loss	miR-34a, miR-29b	Marozzo et al. [86]
	Circulating cell-free mitochondrial DNA (ccf-mtDNA)*	MELAS, MERRF	Elevated during acute episodes; indicates mitochondrial stress	Maresca et al. [70]
Saliva	GDF15	Tested in different PMDs	Detectable and dynamic; correlates with stress, utility might stand in its minimally invasiveness	Huang et al. [55]

Organic acids and acylcarnitines (which could be useful in the differential diagnosis with other metabolic conditions) are not mentioned in the table

*CK* Creatine kinase, *CPEO* Chronic Progressive External Ophthalmoplegia, *FGF21* Fibroblast Growth Factor 21, *GDF15* Growth Differentiation Factor 15, *LHON* Leber Hereditary Optic Neuropathy, *MELAS* Mitochondrial Encephalomyopathy, Lactic Acidosis, and Stroke-like Episodes, *MERRF* Myoclonic Epilepsy with Ragged-Red Fibers, *MNGIE* Mitochondrial Neurogastrointestinal Encephalopathy, *TK2* Thymidine Kinase 2

*Tested also in Cerebrospinal fluid (CSF)

## Neuroimaging for diagnosis and monitoring

Imaging biomarkers provide non-invasive, spatial readouts of tissue bioenergetics of the brain, heart and skeletal muscle, and potentially detect metabolic derangement and structural injury [87–89]. In PMDs, imaging can support diagnosis, map affected organs and pathways, and monitor progression or treatment response as outcome measures in trials. Classic MRI remains key to diagnose Leigh Syndrome with symmetric T2/FLAIR hyper-intensities in basal ganglia, brainstem, and thalamus [90]. Other characteristic MRI patterns affecting deep gray matter nuclei, thalami, brainstem, cerebellum and cerebral white matter frequently guide clinicians toward a PMD and their differential diagnosis [91–94], while CT may sometimes reveal bilateral basal ganglia calcification. Apart from these classical PMDs, it is important to always consider treatable differential diagnoses, that may present with MRI features similar to for example Leigh Syndrome. MRI is also used in the heart to visualize cardiomyopathies and fibrosis [95], which has been reviewed recently elsewhere [96].

Magnetic resonance spectroscopy (MRS) provides non-invasive metabolic information about the biochemical composition of tissues. Proton MRS (^1^H-MRS) has long been used to measure metabolic state, with elevated cerebral lactate representing one of the first biomarkers of mitochondrial dysfunction, for example in MELAS [97] or Kearns–Sayre syndrome/Pearson syndrome, where lactate is increased in damaged white matter regions; and Leigh syndrome, where lactate peaks are seen in gray and white matter alongside high choline levels [98, 99]. The detection of lactate in the brain is more consistent than in serum, correlates with disease activity in several cohorts and may provide a CNS-based indicator of OXPHOS failure [100–102], but cannot necessarily be distinguished from cellular hypoxia seen in other conditions such as seizures or hypoxic-ischemic injuries [103].

Phosphorus MRS (^31^P-MRS) enables non-invasive assessment of cardiac and skeletal muscle bioenergetics. By quantifying high-energy phosphate metabolites, such as phosphocreatine (PCr), inorganic phosphate (Pi), and ATP, ^31^P-MRS allows real-time evaluation of oxidative phosphorylation capacity [104, 105]. Impaired oxidative metabolism typically manifests as prolonged post-exercise PCr recovery [106]. Abnormalities, such as altered phosphate ratios and delayed recovery kinetics, have been demonstrated in patients with mitochondrial myopathies due to mtDNA variants although diagnostic sensitivity is limited [107]. Exercise-based ^31^P-MRS provides a quantitative in vivo measure of skeletal muscle mitochondrial oxidative capacity and has been explored as pharmacodynamic biomarker in preliminary interventional studies [108]. Despite this potential, broader clinical implementation remains limited by the need for specialized hardware, standardized protocols, and expertise in spectral analysis [109, 110] and it is not widely used in longitudinal studies.

Diffusion MRI and arterial spin labeling (ASL) contribute complementary information about microstructure, cytotoxic stress and perfusion, particularly relevant for stroke-like lesions in MELAS, but insufficiently specific for PMDs [111]. Diffusion-weighted MRI (DWI) reveals characteristics abnormalities in MELAS, reflecting metabolic edema rather than vascular ischemia [112]. In Leigh syndrome and Kearns–Sayre syndrome, DWI hyperintensities with abnormal ADC values have also been noted in the brainstem [113].

Molecular PET imaging approaches enhance biomarker sensitivity and specificity, with a potential for directly measuring disease-relevant aspects of mitochondrial biology: brain translocator protein (TSPO) PET scans are typically used to measure neuro-inflammation and microglial activation in a wide range of neurologic conditions [114, 115]. Interestingly, TSPO is an outer mitochondrial membrane protein, and could therefore be affected by impaired mitochondrial function or mass. TSPO PET with the ^11^C-PK11195 ligand appears to show signal changes that correlate with disease severity, and may detect abnormality in absence of MRI changes [116]. Multiple case reports on patients with MELAS indicate that FDG-PET reveals regional glucose hypo- or hyper-metabolism [117, 118].

Challenges persist, in particular for PET, with substantial costs associated with tracer production and imaging. Genetic variants can influence tracer binding [119], there are partial volume effects [120], and mitochondrial dysfunction can vary across tissue types, which together complicate signal interpretation and routine clinical application. Nevertheless, as highlighted in recent reviews [87, 103, 121], many of these imaging approaches may ultimately support earlier diagnosis, stratify disease subtypes, or track treatment response. A summary of the main imaging techniques used in patients with PMDs is presented in Table 3.

**Table 3:** List of the main central nervous system imaging techniques used in patients with Primary Mitochondrial Diseases (PMDs)

Organ	Technique	Sequence	Mitochondrial condition	Observation	References
Brain	MRI	T2/FLAIR	Wide range of PMDs	Symmetric hyper-intensities in white and deep gray matter; Cerebellar atrophy, for example in POLG mutations; Prominent leukoencephalopathy, for example in MNGIE; Symmetric hyper-intensity of basal ganglia, for example in Leigh Syndrome; Focal gray matter lesions, for example in stroke-like episodes caused by MELAS, typically in temporal, parietal, and occipital lobes; Developmental abnormalities, for example in Pyruvate Dehydrogenase deficiency, etc	Extensively reviewed in Saneto et al. [88]
		DWI	MELAS (stroke-like episodes)	Stroke-like lesions typically demonstrate normal or increased apparent diffusion coefficient (ADC) values, indicative of vasogenic edema, rather than restricted diffusion in ischemic stroke	Oppenheim et al. [112]
		Arterial Spin Labeling (ASL)	MELAS (stroke-like episodes)	Peripheral hyper-perfusion observed in stroke-like lesions assists in differentiating from acute ischemic stroke	Li et al. [111]
	MRS	^1^H-MRS	MELAS	An elevated lactate peak and reduced N-acetyl-aspartate (NAA) constitute a metabolic profile indicative of mitochondrial dysfunction	Abe et al. [100]
			Kearns–Sayre syndrome/Pearson syndrome	An increase in lactate within the affected white matter	Kapeller et al. [99]
			Leigh syndrome	Elevated lactate is detected in both affected gray and white matter, often accompanied by increased choline levels	Sijens et al. [98]
		^31^P-MRS	Mitochondrial myopathies	Prolonged post-exercise Phosphocreatine (PCr) recovery	Jeppesen et al. [107]
	PET	TSPO PET	Tested in a cohort of different PMDs	Regional alterations in TSPO ([^11^C]PK11195) binding may correspond to phenotypes or clinical severity	van den Ameele et al. [116]
		FDG-PET	MELAS (stroke-like episodes)	Regional hypo-metabolism or hyper-metabolism, dependent on disease stage or presence of seizures	Liu et al. [117]
Eye	MRI		CPEO	Marked atrophy of the extraocular muscles	Yu-Wai-Man et al. [89]

*CPEO* Chronic Progressive External Ophthalmoplegia, *DWI* Diffusion-Weighted Imaging, *FDG* Fluorodeoxyglucose, *FLAIR* Fluid-Attenuated Inversion Recovery, *MELAS* Mitochondrial Encephalomyopathy, Lactic Acidosis, and Stroke-like Episodes, *MRI* Magnetic Resonance Imaging, *MRS* Magnetic Resonance Spectroscopy, *PET* Positron Emission Tomography, *POLG* DNA Polymerase, Gamma, TS*P*O Translocator protein

## Emerging non-invasive biomarkers and imaging approaches with promise in PMDs

Beyond traditional serum and neuroimaging markers, several innovative methodologies are emerging with the potential to provide sensitive, real-time readouts of mitochondrial dysfunction, metabolic capacity, and tissue-level bioenergetics. These approaches may offer a viable path toward objective stratification and functional endpoints for upcoming clinical trials, although many are still constrained to preclinical models or other diseases and have not been tested in patients with PMDs.

Among laboratory biomarkers, circulating axonal cytoskeletal proteins such as peripherin have emerged as promising biomarkers of peripheral nervous damage [122, 123], paving the way for PMDs presenting with peripheral neuropathy. Ongoing studies, for example in asymptomatic mtDNA variant carriers for LHON, aim to identify signatures predictive of phenotypic conversion [124].

A wide range of imaging techniques is also emerging, with promise as novel biomarkers in PMDs. Near-Infrared Spectroscopy (NIRS) with vascular occlusion testing provides a non-invasive method to assess muscle oxygenation, hemodynamics, and oxidative metabolism. In pediatric populations with PMDs and neuro-genetic disorders, NIRS-derived parameters have shown sensitivity to impaired OXPHOS in small clinical cohorts of patients with mitochondrial or other neuromuscular disorders, correlating with clinical severity and functional limitations [125]. The portability and tolerability of NIRS could make it attractive for longitudinal clinical monitoring, including in pediatric populations [126].

Other imaging techniques seek to enhance spatial specificity and provide mechanistic insights into metabolic alterations. Chemical Exchange Saturation Transfer (CEST), has demonstrated potential in an Ndufs4 knockout mouse model for mapping intracellular lactate with higher resolution than MRS [127], but has not yet been systematically evaluated in patients. Deuterium Metabolic Imaging (DMI) is an MRI-based technology that provides a dynamic map of glucose utilization and downstream metabolites such as glutamate/glutamine or lactate by tracing deuterated glucose, offering insight into real-time tissue bioenergetics. Though only recently applied in neurological disorders, such as Alzheimer’s disease and glioblastoma [128, 129], DMI holds promise for PMDs by enabling direct assessment of glycolytic flux and OXPHOS that could serve as quantitative biomarkers in trials targeting cerebral energy metabolism [129, 130]. However, studies in PMDs are currently lacking. Another emerging MRI-based technique is Muscle Oxygen Extraction Fraction (OEF), which measures the efficiency of oxygen utilization in muscle and might detect changes in early disease stages [132, 133] although these tracers are currently being evaluated primarily in research settings and have not yet been systematically applied in PMD cohorts. Mitochondrial-targeted tracers for PET imaging, such as ^18^F-BCPP-EF, are in development to enable in vivo quantification of complex I activity [131].

Multi-Spectral Optoacoustic Tomography (MSOT) enables high-resolution real-time visualization of tissue structure and function based on photoacoustic contrast. MSOT allows non-invasive interrogation of physiological parameters linked to mitochondrial function, such as tissue oxygen extraction and microvascular dynamics. Although experience in PMDs remains limited, studies in related neuromuscular diseases as Duchenne Muscular Dystrophy [115], Spinal Muscular Atrophy [134] or Pompe Disease [135], support its potential as a future PMD monitoring or response biomarker. Preclinical studies have demonstrated its feasibility in mouse models for quantification of optoacoustic signatures from metabolically active organs with excellent temporal and spatial resolution [136, 137], ultimately appearing a potential bedside-compatible functional imaging biomarker for tissue-level bioenergetics [138], as demonstrated in a recent exploratory study in patients with m.3243 A>G pathogenic variants [REF].

Finally, Raman spectroscopy of tissue samples or in vivo with fiber-optics may capture protein conformation, metabolic signatures, and structural alterations that precede or accompany muscle pathology. Fiber-optic Raman spectroscopy was shown to differentiate between healthy and diseased muscle in mouse, offering a minimally invasive alternative to standard histopathology [139], and might even be combined with muscle electrophysiology [140].

Overall, while these emerging imaging approaches provide exciting opportunities to interrogate mitochondrial biology in vivo, most remain at an early stage of development. Systematic validation in well-characterized PMD cohorts, standardization of acquisition protocols, and demonstration of reproducibility across centers will be essential before these techniques can be considered robust biomarkers for clinical settings and trials.

## Conclusion

In clinical practice, the management of PMDs often remains challenging, in particular due to marked phenotypic and genetic heterogeneity and fluctuating disease trajectories. To date, no single biomarker has proven sufficiently sensitive, specific, and broadly applicable to meet all the diverse diagnostic and longitudinal needs of these heterogeneous disorders. Traditional imaging, biochemical and functional measures remain essential in routine clinical care, yet their limitations highlight the need for complementary approaches. Recent advances in non-invasive laboratory biomarkers, digital and wearable technologies, and metabolic and molecular imaging, although still requiring validation in many cases, provide clinicians with an expanding toolbox capable of capturing complementary dimensions of mitochondrial dysfunction, tissue injury, and functional impairment. At this stage, rather than replacing traditional measures, these biomarkers should be viewed as adjunct options that can refine diagnostic confidence and support clinical monitoring. They are likely to play an increasingly important role in patient stratification and in providing objective readouts of disease activity and treatment response, particularly in clinical trials.

Looking ahead, validation of partially established biomarkers remains a priority, such as NfL in serum, brain, or muscle MRS imaging, or PET tracers. These should be validated in larger cohorts, together with harmonization of image acquisition protocols, and improved reproducibility of digital health technology endpoints. In parallel, exploratory technologies, including Raman spectroscopy, MSOT, DMI, and novel PET tracers should be progressively translated from preclinical studies into clinical evaluation. Over the medium term, prospective longitudinal studies will be essential to determine biomarker responsiveness, predictive value, and utility as pharmacodynamic endpoints in clinical trials. This should occur alongside efforts to develop disorder- and treatment-specific biomarker profiles, leveraging deep phenotyping and machine learning to inform patient stratification and therapeutic decision-making.

Finally, long-term progress will likely rely on integration of both generic and disorder-specific biomarkers into compound measures, for which large-scale cohorts, deep phenotyping and machine learning approaches may prove useful. Given the distinct pathophysiology and therapeutic strategies of individual syndromic groups, the pursuit of universal biomarkers across all PMDs is likely to remain challenging. Biomarker development should therefore align closely with disease pathogenesis and the key metabolic and biochemical pathways amenable to therapy development, or aim to measure clinical phenotypes with direct relevance for patients and their quality of life [15]. As targeted treatments progress toward clinical application, establishing and harmonizing such multidimensional, disorder- and treatment-specific biomarker profiles will be essential to enable rigorous clinical trials and to advance clinical patient care.

## Data Availability

Not applicable.
